# Suspect asymptomatic lesions: Congenital hypertrophy of the Retinal Pigment Epithelium (CHRPE)

**DOI:** 10.22336/rjo.2021.55

**Published:** 2021

**Authors:** Carolina Serpa Braga, Olívia Moura de Paula Ricardo, Frederico de Miranda Cordeiro, Júlia Maggi Vieira, Fábio Borges Nogueira

**Affiliations:** * Fellowship of Glaucoma, Instituto de Olhos Ciências Médicas, Belo Horizonte, Minas Gerais, Brazil; ** Ophthalmology Resident, Instituto de Olhos Ciências Médicas, Belo Horizonte, Minas Gerais, Brazil; *** Department of Ophthalmology, Hospital Vera Cruz - Belo Horizonte, MG, Brazil

**Keywords:** neoplasms, retina, retinal neoplasms, retinal pigment epithelium

## Abstract

Congenital hypertrophy of the retinal pigment epithelium is a rare benign tumor of the ocular fundus that may vary according to three types. It is frequently asymptomatic and diagnosed during routine ophthalmology exam. This lesion has an important differential diagnosis with retinal disease and may be related to systemic diseases, therefore, it is essential to recognize it and keep the follow-up. In this paper, three different cases of each type of CHRPE were described and documented.

**Abbreviations:** CHRPE = Congenital hypertrophy of the Retinal Pigment Epithelium, RPE = Retinal Pigment Epithelium, CDVA = Corrected Distance Visual Acuity, PIO = Intraocular Pressure, FA = Fluorescein Angiography, FAP = Familial Adenomatous Polyposis, RPEH-FAP = Retinal Pigment Epithelial Hamartomas Associated with Familial Adenomatous Polyposis, OCT = Ocular Coherence Tomography

## Introduction

Congenital hypertrophy of the retinal pigment epithelium (RPE) is a rare benign lesion of the ocular fundus, which is frequently asymptomatic and diagnosed before 30 years old during a routine exam. It occurs due to a proliferation of larger and more numerous cells of the RPE containing larger melanosomes - a combination of cellular hyperplasia and hypertrophy. It is a lesion with well-defined margins, flat or minimally elevated, round, soul lesion, with dark color and cut edges. The lesions may be depigmented in senior patients [**1**-**4**].

Some possible differential diagnosis for these lesions can be malignant retinal diseases. It may also be associated with systemic pathologies that must be recognized. Three different cases of the three CHRPE entities were described and documented along this paper.

## Case report

**Fig. 1:**
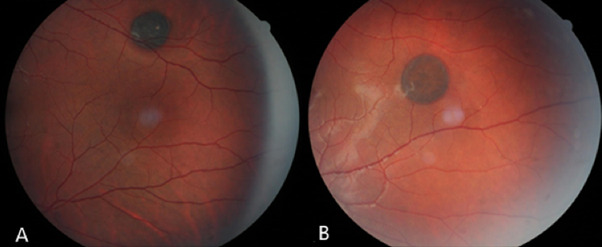
Retinography showing an isolated, pigmented, round and well-delimited lesion in the superior temporal region of the left eye of the first patient **(A)** and temporal in the left eye of the second patient **(B)**

**Fig. 2:**
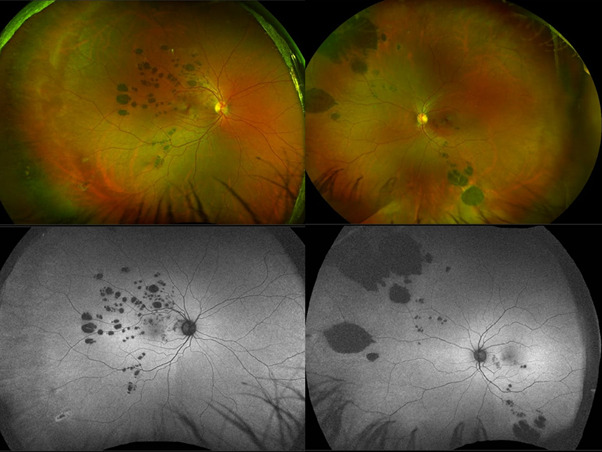
Panfundoscopic retinography showing multiple pigmented, flat lesions, of different sizes, well-delimited in both eyes of the third patient

Three asymptomatic patients were seen during the ophthalmological routine exam. The first one was a 16-year-old healthy male, with no relevant ophthalmology history. The corrected distance visual acuity (CDVA) was 20/ 20 (Snellen) in both eyes; the slit lamp exam and intraocular pressure (PIO) measurement showed no alterations; fundoscopy showed a hyperpigmented lesion of at least 1,5-disc diameter, homogeneous, round, and minimally elevated in the superior temporal region in the left eye (**Fig. 1 A**). The second patient was a 20-year-old healthy female, with no relevant ophthalmology history either. She presented a CDVA of 20/ 20 in both eyes; normal biomicroscopy and PIO measurement; and a fundoscopy with a hyperpigmented lesion, minimally elevated, with well-defined margins in the temporal region of the posterior pole in the left eye (**Fig. 1 B**). The third patient was a 33-year-old healthy woman, with no relevant ophthalmology history, a CDVA of 20/ 20 in both eyes, normal biomicroscopy and tonometry and a fundoscopy with retinal hyperpigmented lesions, multiple, round, well-delimited, flat, and bilateral (**Fig. 2**).

The first two patients presented a fluorescein angiography (FA) that evidenced a defect on the blockage of the RPE. The third patient, however, underwent the autoFA and panfundoscopic retinography. Colonoscopy was requested for all the patients to investigate the Familial Adenomatous Polyposis (FAP) and to see if anyone was compatible. Based on the findings, the condition of Congenital Hypertrophy of the Retinal Pigment Epithelium (CHRPE) could be diagnosed.

## Discussion

The CHRPE is a benign uncommon finding that occurs due to a proliferation of the cells of the pigmented epithelium. It is frequently a flat or minimally elevated pigmented, round, oval lesion, with well-delimited margins that emerge from the periphery of the ocular fundus. A depigmented halo located inside the lesion may commonly appear [**2**]. CHRPE represents a group of disorders that can have three varieties: unifocal CHRPE, multifocal CHRPE (“*bear tracks*”) or atypical multifocal CHRPE [**4**,**5**].

The typical isolated CHRPE represents a flat or minimally elevated pigmented, round or oval, well-delimited lesion that typically appears from the peripheral fundus. The multiple congenital hypertrophies of the RPE (“*Bear Tracks*”) show typically segmented hyperpigmented, well-delimited, flat, dark-grey, or black alterations, which are similar to bear tracks and generally emerge near the optic disc, becoming more numerous and larger at the periphery. They can be uni or bilateral (rare) and are normally not associated with functional consequence [**6**]. The atypical multiple entity is bilateral, has multiple oval, fishtail or comma-shaped lesions, associated with margins and perilesional area, which is irregularly hypopigmented [**5**].

The last type of CHRPE presents a close relation to Familial Adenomatous Polyposis (FAP) and others syndromes such as Gardner and Turcot. Due to this condition, the thorough investigation of the family history, neuroepithelial tumors of the central nervous system and a colon propaedeutic are necessary. To distinguish the entities that are not associated with gastrointestinal malignancy, the term “*retinal pigment epithelial hamartomas associated with familial adenomatous polyposis*” (RPEH-FAP) was created [**5**].

FAP is an autosomal dominant disease with elevated penetrance characterized by the emergency of hundreds to thousands adenomatous polyps frequently during the second decade of life. Its evolution to colorectal carcinoma is considered inevitable in patients not undergoing prophylactic colectomy [**6**,**7**]. Gardner syndrome is an autosomal dominant condition that starts in advanced ages with colorectal polyps, osteomas of mandible and long bones, epidermoid cysts, and other features [**6**,**8**].

Differential diagnosis can be presented by inflammatory, infectious, reactive, or neoplastic chorioretinal lesions, as melanocytic choroidal naevus, melanomas, choroidal melanocytoma, subretinal hematoma, epiretinal membrane, hyperplasia of the retinal pigment epithelium (RPE), adenoma or adenocarcinoma of the RPE, retinal and choroidal hamartoma [**2**,**6**,**9**].

The ultrasound and fluorescein angiography (FA) are typically used to distinguish the hypertrophy of the RPE from the uveal melanoma and rare intraocular tumors [**1**]. The ultrasound frequently evidences that the RPE hypertrophy is flat or minimally elevated and slightly hyperreflexive [**1**,**6**]. The FA shows hypofluorescence, except for the atrophy areas, which are hyperfluorescent [**1**-**3**]. The lesions are hypoautofluorescencent in the autoFA [**3**]. In the ocular coherence tomography (OCT), the overlying retina is thinned and the retinal pigment epithelium can be thickened or thinned. The choroid has normal thickness and vascular appearance underlying the CHRPE compared to the immediate tissue outside the lesion margin [**8**].

The CHRPE are benign lesions that are generally diagnosed accidently during an ophthalmology routine exam, being necessary to know how to distinguish them from the threatening injuries [**6**]. Any treatment is necessary, but the follow-up is essential because if there is an increase in size or an appearance of a protruding nodule, it can be an adenoma or adenocarcinoma, and a few cases can develop a choroidal neovascular membrane above the lesions [**2**,**3**,**6**]. In addition, the investigation of FAP is essential in patients with ocular findings, especially in the atypical variety.

## Conclusion

The CHRPE is a benign rare finding diagnosed occasionally during a routine exam that needs to be known by the ophthalmologists. In the most cases, any treatment is necessary, however, is important to distinguish it from other pigmented suspect lesions. 

**Conflict of interest**

The authors declare no conflicts of interest.

**Informed Consent and Human and Animal Rights statements**

Informed consent has been obtained from all individuals included in this study.

**Authorization for the use of human subjects**

Ethical approval: The research related to human use complies with all the relevant national regulations, institutional policies, is in accordance with the tenets of the Helsinki Declaration, and has been approved by the Ethics Committee of Faculda de Ciências Médicas, Belo Horizonte/MG, Brazil.

**Acknowledgements**

None.

**Sources of funding**

All authors had no financial disclosures or support in this work. The authors declare that their research has not been funded by any entity.

**Disclosures**

None.
